# A New Guiding Suturing Technique for Reshaping of the Antihelix in Patients with Prominent Ears

**DOI:** 10.1007/s00266-024-04478-0

**Published:** 2024-11-22

**Authors:** Ayman Altramsy, Asmaa Ali Dahy, Amany Attalah Gad, Ahmed Abu-Elsoud, Rania Fouad Khattab, Ahmed Mamdouh Nafeh, Rasheda Azzam, Ali Mohamed Elameen

**Affiliations:** 1 Department of Plastic and Reconstructive Surgery, Faculty of Medicine For Girls, Al-Azhar University, Gameat Al Azhar, Nasr City, Cairo, Egypt; 2 Department of Plastic and Reconstructive Surgery, Faculty of Medicine (Assiut branch), Al-Azhar University, Cairo, Egypt; 3 Department of Plastic and Reconstructive Surgery, Faculty of Medicine, Helwan University, Cairo, Egypt; 4 Department of Anesthesia and Intensive Care, Faculty of Medicine For Girls, Al-Azhar University, Cairo, Egypt; 5 Department of Plastic and Reconstructive Surgery, El-Sahel Teaching Hospital, Cairo, Egypt

**Keywords:** Otoplasty, Prominent ears, Guiding sutures, Mustardé

## Abstract

**Background:**

Prominent ears are the most common congenital anomaly of the head and neck. A complete understanding of the definition of prominent ears is necessary. The present retrospective study described guiding sutures to hold the antihelix in a temporary corrected position before placing the permanent Mustardé sutures.

**Methods:**

This study was performed between January 2021 and February 2023. All patients with prominent ear deformities subjected to guiding sutures and Mustardé-based otoplasty were included. The surgical-related outcomes and surgeons’ satisfaction were evaluated. The patients’ satisfaction and health-related quality of life were reported.

**Results:**

The current study included 60 patients with prominent ear deformities. There were 34 (56.66%) males and 26 (43.33%) females with a mean age of 12.2±7.8 years. The mean total operative time was 49±22 minutes. Five (8.33%) patients had suture extrusion, and no case of asymmetry, recurrence, or skin necrosis was documented. There were 55 (91.66%) patients satisfied with the final appearance of their ears, and five (8.33%) patients were not satisfied. The mean general health subscale was 57.1±6.9, and the mean physical health subscale was 8.7±1.5.

**Conclusions:**

The guiding sutures allowed easy accessibility for reshaping the antihelix in patients with prominent ears. These sutures allowed a relatively shorter operative time, and stable reshaping of the antihelix allowed for a shorter recovery time. This was associated with a low complication rate with no asymmetry, recurrence, or revision surgery. Patients operated on under local anesthesia achieved shorter operative time and better pain control.

**Level of Evidence III:**

This journal requires that authors assign a level of evidence to each article. For a full description of these Evidence-Based Medicine ratings, please refer to the Table of Contents or the online Instructions to Authors www.springer.com/00266.

**Supplementary Information:**

The online version contains supplementary material available at 10.1007/s00266-024-04478-0.

## Introduction

Prominent ears are the most common congenital anomaly of the head and neck. It accounts for approximately 5% of the general population [1, 2]. This facial deformity is inherited and potentially results from the antihelix’s underdevelopment and increased the conchoscaphal angle and conchal hypertrophy. This deformity negatively impacts the psychological and social well-being of the patients. Patients frequently experience low self-esteem, emotional trauma, negative body image, anxiety, and social phobia [3]. Otoplasty is the most common performed cosmetic procedure in children. It restores acceptable conchoscaphalic, auriculocephalic, and conchomastoidal angles and achieves symmetrical aesthetic auricles [4, 5]. The most substantial aim of otoplasty is to increase the patient’s satisfaction rate and to improve their psychological well-being [6]. The treatment plan should be constructed to meet the patient’s needs and to achieve the desired aesthetic and functional outcomes [7].

Numerous techniques have been described for correcting prominent ears. These techniques are either cartilage sparing (suture-based) or cartilage cutting. The latter techniques are associated with a higher risk of irregular contours, restricting their frequent application in clinical practice [8]. Suture-based techniques reshape the antihelix and set back the concha without leaving any surgical marks on the front. These techniques reduce the damage to the perichondrium and the risk of cartilage necrosis associated with cartilage scoring [9]. The Mustardé suture technique accentuates the antihelical fold with horizontal mattress sutures without cartilage incision. Despite the acceptance of this method, it is associated with a higher risk of suture extrusion, infection, and recurrence, with an overall complication rate of up to 24% [10, 11]. Furthermore, the length, orientation, and location of the horizontal Mustardé sutures may alter the morphological features of the recreated antihelix and, subsequently, the final cosmetic results. The inappropriate placement of sutures may otherwise result in an unnatural and unpleasant morphology of the antihelix. This may result in unsatisfactory and asymmetrical unfavorable results after otoplasty. This highlighted the need to decide where to position the correct sutures and where to incise the cartilage precisely before placement of the permanent sutures [9, 12].

A complete understanding of the definition of prominent ears is necessary and should be comprehensively evaluated [13, 14]. The outcomes of otoplasty are defined by the native anatomy, the surgical procedure, and the attention to patient-centered outcomes [15]. The literature is inconclusive regarding the precise position of the sutures-based techniques for otoplasty, with considerable variation in the definition of aesthetically acceptable ears [16]. Therefore, the present retrospective study described guiding sutures to hold the antihelix in a corrected position before the permanent Mustardé sutures are placed. This could help to precisely identify the position of the permanent Mustardé sutures to achieve precise control of antihelix and auricular shape. These guiding sutures could provide a reliable cartilage fixation approach, holding the antihelix in a temporary corrected position before placing the permanent Mustardé sutures. Furthermore, these guiding sutures may offer easy accessibility for reshaping the antihelix and could minimize the number of sutures performed beneath the posterior skin and reduce sutures-related complications.

## Methodology

The current study followed the ethical recommendations of the Ethics Unit, Faculty of Medicine for Girls, Al-Azhar University, Cairo, Egypt. The potential benefits and risks of otoplasty were explained to all patients before the surgery. The study was based on the ethics reported in the Declaration of Helsinki [17]. The present study was performed following the Strengthening Reporting of Observational Studies in Epidemiology (STROBE) Statement guidelines for conducting retrospective studies [18] (Supplementary Table 1).

## Study Design

This retrospective study was performed between January 2020 and February 2023. The study was conducted at the Plastic and Reconstructive Surgery Department at Al-Zahraa University Hospital, Faculty of Medicine (Girls), Al-Azhar University, Cairo, Egypt.

## Eligibility Criteria

All patients with prominent ear deformities subjected to guiding sutures and Mustardé-based otoplasty were included. Patients treated with cartilage-scoring techniques or patients who underwent multiple operations were excluded. Patients lost to follow-up or patients with less than six months of postoperative follow-up were excluded.

## Surgical Procedure

The procedures were conducted under entirely aseptic conditions using general or local anesthesia. Non-compliant patients and children were operated on under general anesthesia, while adult-compliant patients were operated on under local anesthesia. The local anesthetic solution is composed of 2% lidocaine with 1:100,000 adrenaline. The lidocaine was partially mixed with bupivacaine 1%. The anesthetic solution was injected using a 30-gauge needle with a total amount of 7 mg/kg body weight. An average dosage of 13±2 ml of the anesthetic solution was injected with strict precautions to ensure extravascular injection. Continuous blood pressure and cardiac monitoring were performed. The anesthetic solution infiltrated around the ear as a ring block as well as directly throughout the proposed incisions and dissection planes. Direct injection starts in the subcutaneous tissue of the posterior surface of the ear, followed by advancing the needle to the anterior surface through the cartilage to place the anesthetic solution under the skin layer. The posterior and inferior aspects of the external auditory canal were injected with the anesthetic solution to anesthetize the Arnold branch of the vagus nerve [19–21].

The patients were positioned to maintain bilateral exposure of the ears. The antihelix was temporarily reshaped with gentle digital pressure to achieve the desired degree of correction. This degree of correction was adjusted with respect to the patients and the surgeons’ preferences. This position was maintained by two horizontal mattress guiding sutures (Proline 3-0) passing from the skin at the anterior side to the posterior side through the antihelix fold. The first suture was located perpendicularly at the most protruding part of the antihelix, which was supposed to withstand the major force of cartilage rebound. The second suture was situated at the superior or inferior antihelical crus bifurcation or slightly more superior, maintaining the shape of antihelical crus. The suture knots were tied in an appropriate tension from the lower to the upper direction to maintain the naturally shaped antihelix. Photographs of the front and side views of the bilateral ears were captured for intraoperative reference.

**Fig. 1:**
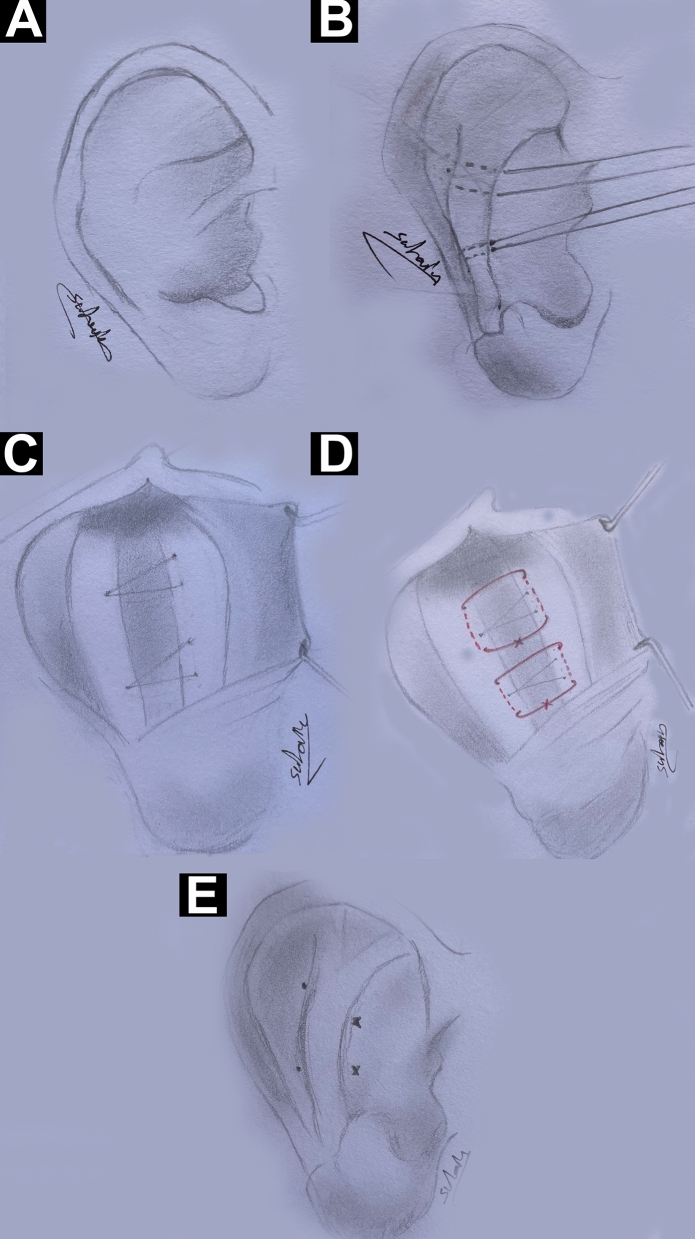
Drawings of the guiding and Mustardé sutures. **A** The prominent ear with ill-defined antihelix. **B** The two guiding sutures were placed to maintain the shape of both antihelical crus before the placement of the permanent Mustardé sutures. **C** The location of the guiding sutures on the posterior aspect of the auricle located perpendicular to the antihelix. **D** Placement of the permanent Mustardé sutures at the level of guiding sutures **E** The shape of the antihelix after placement of the guiding and Mustardé sutures

**Fig. 2:**
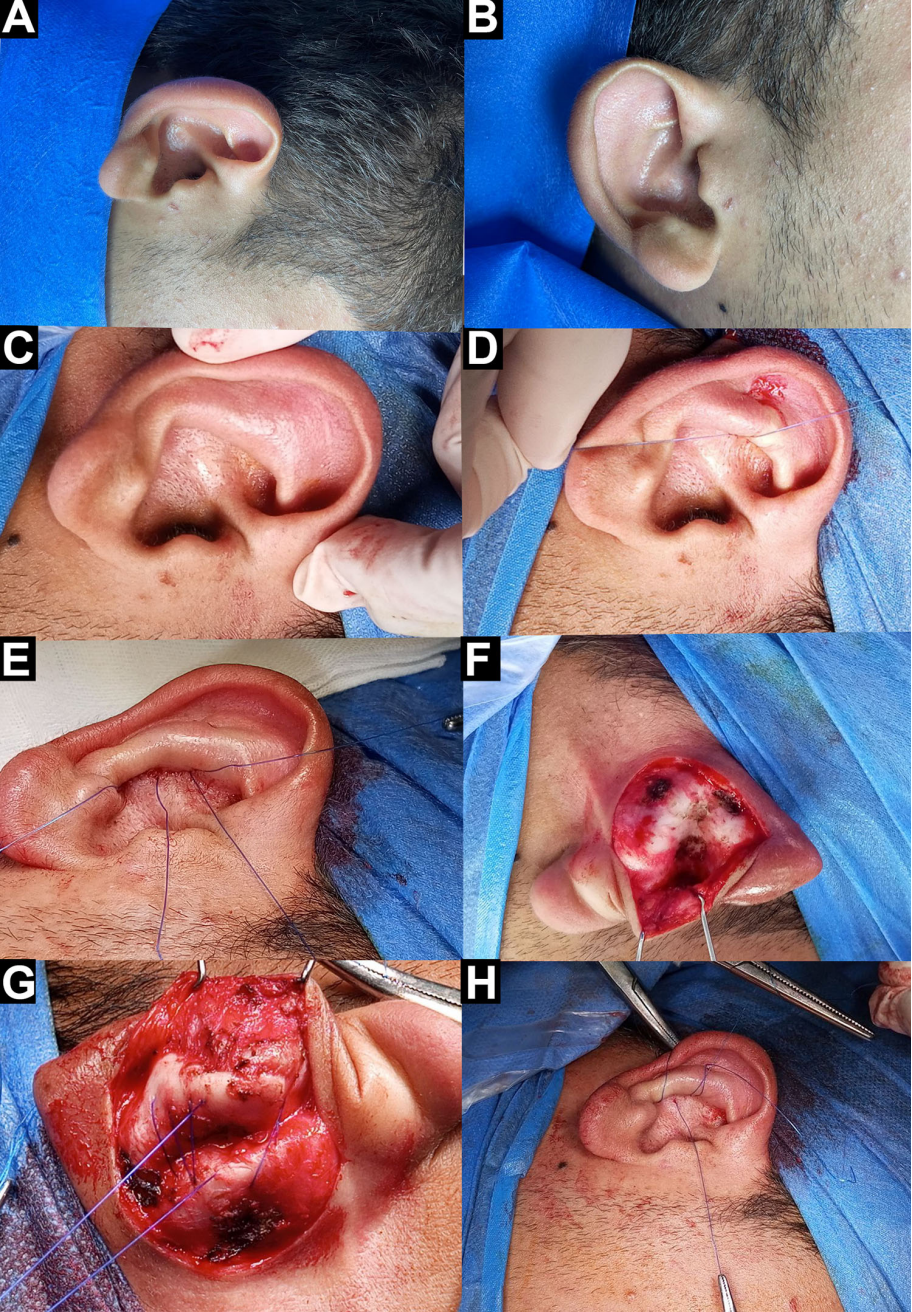
A 18-year-old male patient presented with bilateral prominent ears**. A** and **B** Lateral and side views of the ears just preoperatively. **C** The antihelix was temporarily reshaped with gentle digital pressure to preliminary achieving of the desired degree of correction. **D** The first guiding suture was located at the most protruding point of the antihelix. **E** The second guiding suture was positioned to maintain the shape of both antihelical crus, and the guiding sutures knots were tied in an appropriate tension. **F** Incision of the skin at the posterior aspect of the pinna and exposure of the auricular cartilage. **G** The Mustardé sutures were placed passing from the nadir of the scapha or fossa triangularis to the opposite nadir of the conchal cartilage. **H** Immediate postoperative shape and position of the architectures of the ear

Local anesthesia was infiltrated in the post-auricular sulcus and post-auricular pinna. Incision of the skin at the posterior aspect of the pinna was performed, and the posterior aspect of the auricular cartilage was exposed, allowing inferior access to the conchal bowel. Dissection was executed superiorly to allow sufficient cartilage exposure in the upper helix and triangular fossa. The Mustardé sutures were placed passing from the nadir of the scapha or fossa triangularis to the opposite nadir of the conchal cartilage. This was performed using two or more Proline (3-0) conchoscaphal or concho-fossa triangularis horizontal mattress sutures based on the patient’s characteristics and the surgeon’s preference. The sutures were placed and secured under appropriate tension based on the guiding sutures performed preoperatively. The knots of the Mustardé sutures were buried in the created fold of antihelix. The skin was closed with Proline (4-0) running sutures. The guiding sutures were left in place for three weeks postoperatively. The head was wrapped for the first week, and a protective headband was worn for seven days postoperatively. Postoperative consultations were scheduled weekly for a month, then bimonthly for six months, and then monthly for a year (Figs. 1 and 2 and Supplementary Video 1)

## Data Collection and Study Endpoints

The data were collected retrospectively in a well-structured Excel sheet. The data included patients’ demographics, such as age, sex, comorbidities, and previous surgeries. The operative-related data were collected, including laterality of the surgery, age at operation, anesthesia type, suture materials, number of guiding sutures, number of Mustardé sutures, and operative time. The surgical-related outcomes were collected, including the suture and scar-related complications. The surgeons’ satisfaction was based on the residual prominence, the symmetry of the ears, and the complications. Patient and family satisfaction was evaluated based on the shape, prominence, and symmetry of the ears. The Arabic version of the Glasgow benefit inventory (GBI) questionnaire was used to assess the health-related quality of life changes after otoplasty [22]. The questionnaire was divided into four subscales. This included the general health subscale, physical health subscale, social support subscale, and GBI total score.

The pain was assessed immediately postoperative and at discharge. The visual analog scale (VAS) was used to determine the severity of postoperative pain. It is a 10-point scale, with the left side revealing no pain with a smiling face, while the right side shows the worst pain ever with a frowning look [23]. None of the patients had analgesics prescribed immediately after the surgery. If any patient mentioned substantial pain, they advised taking Ibuprofen 600mg every 6 hours until the pain disappeared. Patients allergic to non-steroidal anti-inflammatories were advised to take the safe dosage of paracetamol.

## Statistical Analysis

Continuous normally distributed data were reported in the form of mean and standard deviation. Non-normally distributed data were reported using median and range. Categorical variables were expressed in the form of the number, and percentage. Non-normally distributed data were reported using median and range, and related groups were compared using the Kruskal–Wallis test. Statistical analysis was performed using SPSS software version 25 for Windows (SPSS Inc., Chicago, IL, USA) [24]. Figures were renovated using GraphPad Prism (GraphPad Software, Inc, San Diego) software version 8 [25].

## Results

A total of 68 patients were assessed for eligibility for the current study. Eight patients with a history of previous otoplasties were excluded. Sixteen patients with prominent ear deformities were finally included for the analysis with a mean age of 12.2±7.8 years. There were 34 (56.66%) males and 26 (43.33%) females, and five (8.33%) patients had syndromic prominent ears. There were 12 (20%) patients with right prominent ear and 9 (15%) patients with left prominent ears, while 39 (65%) patients had bilateral prominent ears. Otoplasties were performed under general anesthesia for 44 (73.33%) patients and under local anesthesia for 16 (26.66%) patients. The mean total operative time was 49±22 minutes with a relatively shorter duration among unilateral cases than bilateral cases with a duration of 35±8.5 and 57±9.8 minutes, respectively. The mean operative time with local anesthesia was 39±4 minutes and 53±8 minutes with general anesthesia. The number of guiding sutures was two horizontal mattress sutures (Table 1).

**Table 1:** Baseline demographic characteristics and surgery-related data

Variables	Mean± SD/ Number (%)
*Patient related data*	
*Age (Years)*	12.2±7.8
*Gender*	
Males	34 (56.66%)
Females	26 (43.33%)
Syndromic prominent ears	5 (8.33%)
*Laterality*	
Unilateral	
Right side	12 (20%)
Left side	9 (15%)
Bilateral	39 (65%)
*Surgery related data*	
*Anesthesia*	
General anesthesia	44 (73.33%)
Local anesthesia	16 (26.66%)
Total operative time (Minutes)	49±22
Unilateral cases	35±8.5
Bilateral cases	57±9.8
Local anesthesia	39±4
General Anesthesia	53±8
Number of guiding sutures	2

Abbreviations; *SD*: Standard deviation

## Outcomes of Guiding Sutures

**Fig. 3:**
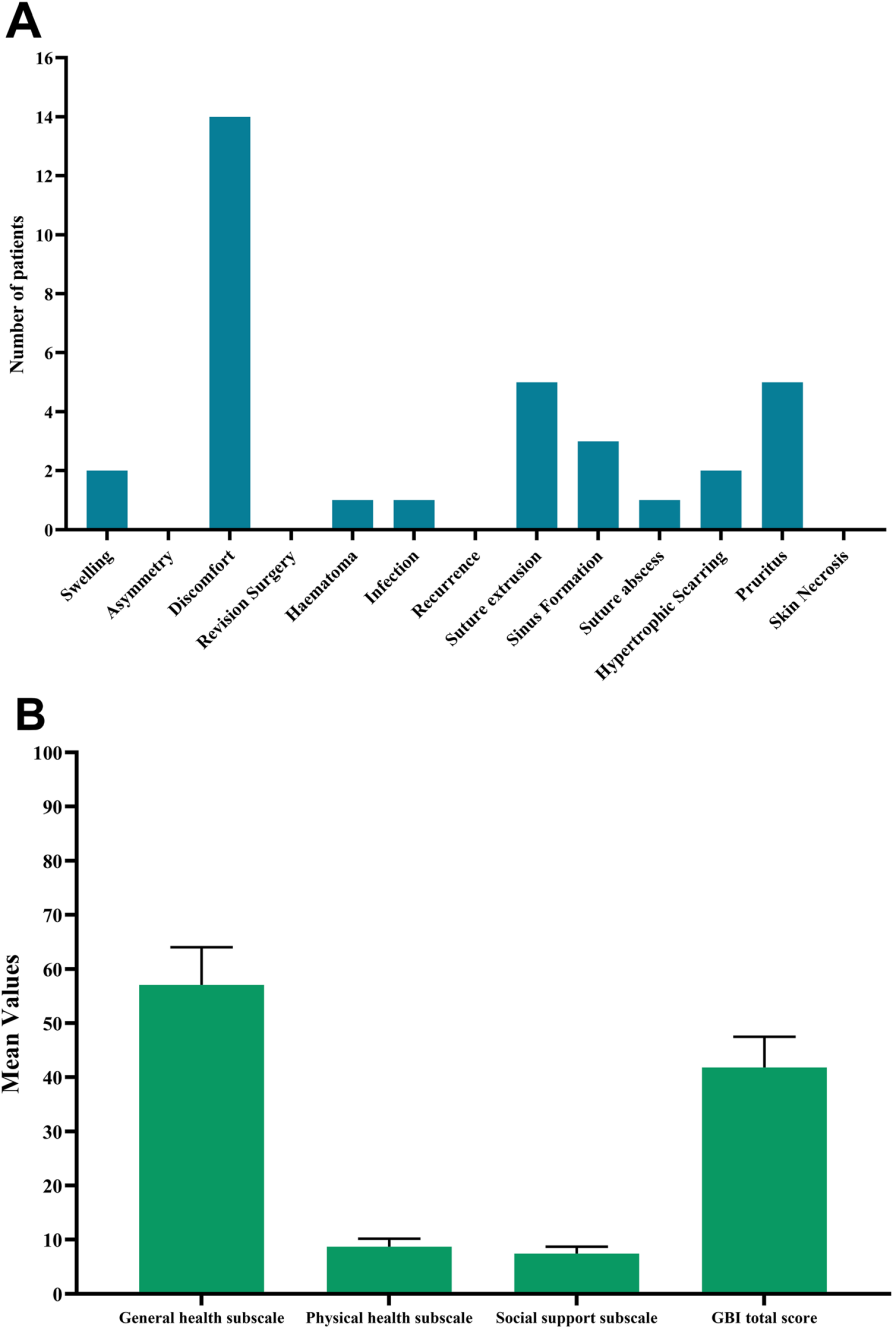
**A** Bar chart showing the pattern of postoperative complications after otoplasty with guiding sutures for patients with prominent ears. **B** Error bar chart showing the mean values of Glasgow benefit inventory (GBI) questionnaire

Two (3.33%) patients had postoperative swelling. Fourteen (23.33%) patients complained of postoperative discomfort that was relieved a few days after the surgery. One patient experienced hematoma and another patient had a surgical site infection. Five (8.33%) patients had suture extrusion and 5 (8.33%) patients had pruritus at the site of the guiding sutures. No case of asymmetry, recurrence, or skin necrosis was documented. No revision operations were performed for the included patients. The mean duration of hospital stays was 11.8±2.7 hours (Table 2 and Fig. 3A).

**Table 2:** Surgical and satisfaction outcomes of otoplasties

Variables	Mean± SD/ Number (%)
*Complications*	
Swelling	2 (3.33%)
Asymmetry	0 (0%)
Discomfort	14 (23.33%)
Revision surgery	0 (0%)
Hematoma	1 (1.66%)
Infection	1 (1.66%)
Recurrence	0 (0%)
Suture extrusion	5 (8.33%)
Suture abscess	1 (1.66%)
Hypertrophic scarring	2 (3.33%)
Pruritus	5 (8.33%)
Skin necrosis	0 (0%)
*Hospital stays (hours)*	11.8±2.7
*Functional outcomes*	
*Patients satisfaction*	
Satisfied	55 (91.66%)
Not satisfied	5(8.33%)
*Surgeons satisfaction*	
Satisfied	52 (86.66%)
Not satisfied	8 (13.33%)
Follow-up time (months)	8.2±1.9
*Glasgow benefit inventory (GBI) questionnaire*	
General health subscale	57.1±6.9
Physical health subscale	8.7±1.5
Social support subscale	7.4±1.3
GBI total score	41.8±5.7

Abbreviations; *SD*: Standard deviation

**Fig. 4:**
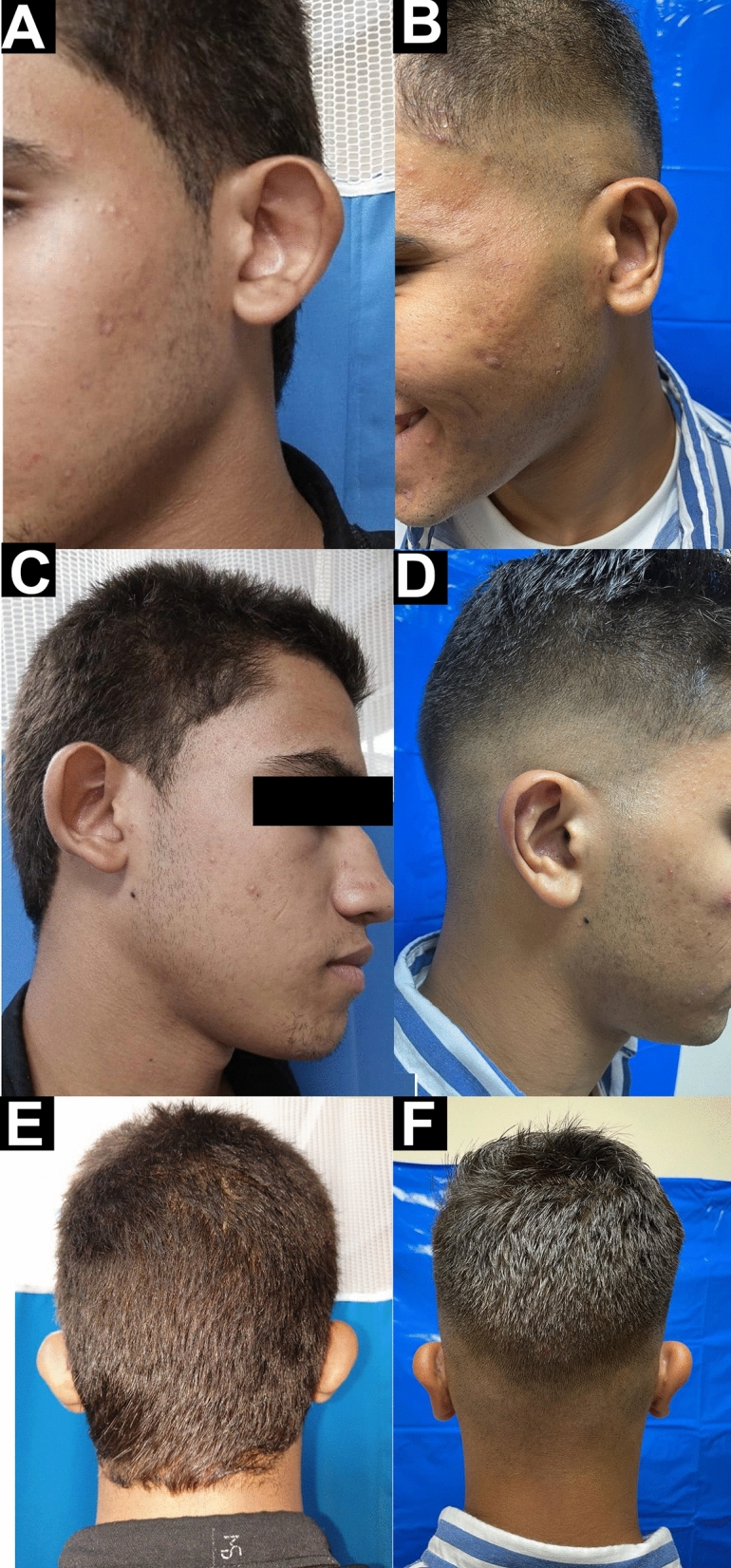
A 18-year-old male patient presented with bilateral prominent ears. **A** Preoperative lateral view of the left ear. **B** Postoperative lateral view of the left ear showing the position of the helix and the distance between the ear and the mastoid process. **C** Preoperative lateral view of the right ear. **D** Postoperative lateral view of the right ear showing the position of the helix and the distance between the ear and the mastoid process. **E** Preoperative posterior view. **F** Postoperative posterior view showing the shape and position of the architectures of the ear. (Postoperative figures eight months postoperatively)

There were 55 (91.66%) patients satisfied with the final appearance of their ears and five (8.33%) patients were not satisfied. There were 52 (86.66%) surgeons satisfied with the outcomes and eight (13.33%) were not satisfied. The mean general health subscale of the GBI scale was 57.1±6.9, and the mean physical health subscale was 8.7±1.5. The mean social support subscale was 7.4±1.3, and the mean GBI total score was 41.8±5.7. The mean follow-up period was 8.2±1.9 months (Table 2 and Figs. 3B, 4, and Supplementary Fig. 1).

The median immediate postoperative VAS was significantly low among the local anesthesia group (*P*=0.036). Four (25%) patients experienced no pain with local anesthesia, while 3 (6.81%) patients experienced no pain after general anesthesia. Fourteen (31.81%) patients had very severe pain after general anesthesia, whereas two (4.54) patients had the worst pain possible. Nine (56.25%) patients had mild pain after local anesthesia, while 17 (38.63%) had severe pain after general anesthesia (*P*=0.087). At discharge, there was no significant difference between local and general anesthesia patients with a median VAS score of 0 (0-3) and 3 (0-6), respectively (*P*=0.69). The median number of analgesics was significantly low among the local anesthesia group, with a median of 0 (0-2) and a *P*-value of 0.031 (Table 3).

**Table 3:** Postoperative pain and analgesic usage

Variables	Local Anesthesia with adrenaline (*n*=16)	General anesthesia (*n*=44)	*P*-Value
	Median (Range)/Number (%)	Median (Range)/Number (%)	
*Immediately postoperative*			
Visual analogue scale	2(0-5)	5 (2-9)	0.036
No pain	4 (25%)	3 (6.81%)	0.087
Mild	9 (56.25%)	2 (4.54%)	
Moderate	2 (12.5%)	6 (13.63%)	
Severe	1 (6.25%)	17 (38.63%)	
Very severe	0 (0%)	14 (31.81%)	
Worst pain possible	0 (0%)	2 (4.54%)	
*At Discharge*			
Visual analogue scale	0 (0-3)	3 (0-6)	0.69
No pain	10	4 (9.09%)	0.48
Mild	5	9 (20.45%)	
Moderate	1 (6.25%)	11 (25%)	
Severe	0 (0%)	13 (29.54%)	
Very severe	0 (0%)	7 (15.9%)	
Worst pain possible	0 (0%)	0 (0%)	
*Number of taken analgesics*	0 (0-2)	2 (1-6)	0.031

Abbreviations; *P*: Probability value

## Discussion

Numerous techniques have been described for correcting prominent ears. The most common procedure is suture-based, which aims to recreate the antihelical fold and set back the concha. There is a paucity of evidence regarding the accurate position of the Mustardé sutures [26]. The present study introduced a simple method that guides surgeons to accurately decide where to position the Mustardé sutures while holding the ears in a temporarily corrected position. These guiding sutures avoid the need for ink use, which often smudges in the operative field [27]. The current study highlighted the safety, feasibility, and effectiveness of guiding sutures for reshaping the antihelix in patients with prominent ears. The guiding sutures provided easy accessibility for defining the antihelix in a relatively shorter operative time. The technique was associated with a low complication rate with no asymmetry, recurrence, or revision surgery. Most patients were satisfied with their ears’ final appearance and reported improved physical and social well-being and quality of life. The guiding suture is a valuable addition to the armamentarium of otoplasty, guiding the surgeons to precisely define the architectures of the ears promptly and shallow the learning curve for junior surgeons.

The otoplasty for prominent ear correction aims to restore the antihelix’s ideal shape and achieve bilateral symmetry. Previous methods have been described to aid the precise placement of Mustardé sutures. Hilger et al.,1997 described contouring sutures that allowed the surgeon to fold the ear forwards, expose the post-auricular incision, and place permanent Mustardé sutures. These contouring sutures maintain the cartilage in a predetermined position while the ear cartilage is manipulated [28]. While this modification fixes the antihelix in a stable position while placing the permanent sutures, placing three mattress sutures and removal of these contouring sutures immediately after the surgery may be associated with long-term adverse effects that had not been studied in Hilger et al.,1997 study. Mathur et al., 2010 described three hypodermic needles to precisely place sutures in otoplasty [29]. The needles were placed at the lower, middle, and upper poles, passing from the helical side to the scapha. However, these needles offer rigid temporary correction of the antihelix, limiting the ability to manipulate the antihelix during otoplasty. This highlights the need for a more reliable technique guiding the placement of permanent sutures in otoplasty.

In the present study, all patients achieved the desired symmetry and natural shape of the antihelix with no case of recurrence. Suture-based techniques are considered prone to recurrence and asymmetry. Alanazi et al., 2024 reported a risk of recurrence and reoperation of 4.27% after cartilage-sparing otoplasty, being the most common complication [30]. Placement of horizontal sutures of Mustardé technique may allow the knots to squeeze the antihelix along the apex with multiple forces applied along the axial direction of the antihelix. These forces interact with each other, resulting in distortion of the natural curvature of the antihelix and asymmetry. The guiding sutures were applied perpendicular to the curvature of the antihelix. The placement of two mattresses guiding sutures allows the fixation forces to be eventually distributed along the antihelix while placing the Mustardé sutures. Furthermore, the increasing length of the cartilage being fixed with the perpendicular placement of the guiding sutures prevented the minimal curvature of the antihelix, enabling firm fixation and a reduced risk of recurrence [31]. Recurrence occurs due to the imbalance between the strength provided by fixation and the intrinsic resilience of the auricular cartilage. The shape of the cartilage depends on adequate suture fixation during the postoperative period until scar adhesions become firm. Placement of the guiding sutures for three weeks postoperatively may provide more adhesions in a timely period.

The guiding sutures reduced the operative time for unilateral and bilateral cases with protruding ears. Placement of the guiding sutures defines the antihelical fold, allowing quicker and more precise placement of the permanent Mustardé sutures. The guiding sutures reduced the operative time by approximately 15 and 33 minutes for unilateral and bilateral cases relative to the time reported in Boroditsky et al., 2021 [32]. Furthermore, guiding sutures decreased the need for prolonged head bandages without risk of recurrence or asymmetry during the follow-up period. These guiding sutures acted as a tie-over that reduced the risk of hematomata formation and desiccation of tissues. Guiding sutures provided improved surgical outcomes and a faster recovery period for patients with prominent ears.

Otoplasty using guiding sutures is an effective method when evaluating the patient’s satisfaction and improvement of the health-related quality of life. This effectiveness also relies on the objective parameters determined by the surgeons, with a high surgeon satisfaction rate. Parallel with these findings, Braun et al., 2010 reported a significant increase in the patient’s quality of life scores with a high satisfaction rate after suture-based otoplasty [33]. Choi et al., 2017 reported a long-lasting improvement in health-related quality of life after otoplasty using Mustardé suture technique and conchomastoid suture techniques for the protruding ears [34]. However, these advantages should be considered in the context of adverse effects associated with suture-based otoplasty. This includes suture extrusion, sinus formation, and abscess formation. In the present study, these complications were limited and were comparable to the risk of complications reported in the literature. Boroditsky et al., 2020 reported a similar rate of suture extrusion, abscess, and hematoma after Mustardé otoplasty for prominent ear deformities [32]. These complications were handled successfully in all cases without adversely impacting patient and surgeon satisfaction outcomes.

The present study highlighted the safety and feasibility of guiding sutures in different settings. The technique can be implemented as an office-based procedure under local anesthesia with shorter operative time and better pain control relative to general anesthesia. In this respect, Hazkour et al., 2023 reported the safety and feasibility of otoplasty under local anesthesia for the pediatric population with a reduced parental anxiety score and similar satisfaction rate in contrast to general anesthesia. However, local anesthesia was associated with a higher risk of conchal bowl hematomas and recurrence [35]. The guiding sutures overcome the risk of recurrence with no revision surgery documented in the present study. The combination of lidocaine, adrenaline, and bupivacaine increases the duration of anesthesia and reduces the needed analgesics. Otoplasty under local anesthesia is a cost-saving procedure, offering safer and equally effective ear surgery with the elimination of the risks associated with general anesthesia [36].

The present study introduced guiding sutures for reshaping the antihelix in patients with prominent ears. These sutures aid surgeons in determining the placement of Mustardé sutures precisely. However, the study has some limitations that need to be considered. The retrospective nature of the study conveys inherent limitations, including the risk of selection, reporting, and information biases. There was no comparative arm to validate the outcomes of guiding sutures with the available conventional techniques. Further randomized controlled trials with prolonged follow-up periods are needed to mitigate the current study’s limitations.

## Conclusions

The guiding sutures allowed easy accessibility for reshaping the antihelix in patients with prominent ears. These sutures precisely defined the auricular architectures before placing permanent Mustardé sutures. The guiding sutures allowed a relatively shorter operative time, and stable reshaping of the antihelix allowed for a shorter recovery time. This was associated with a low complication rate with no asymmetry, recurrence, or revision surgery. The guiding sutures could shallow the learning curve of otoplasty for plastic surgeons with considerable patient’ and surgeon satisfaction and improved health-related quality of life. Patients operated on under local anesthesia with adrenaline achieved shorter operative time and better pain control relative to patients operated on under general anesthesia.

## Supplementary Information

Below is the link to the electronic supplementary material.

Supplementary file1 (DOCX 384 KB)

Supplementary file2 (DOCX 33 KB)

Supplementary file3 (MP4 159328 KB)
