# Supplementary material for: A New Guiding Suturing Technique for Reshaping of the Antihelix in Patients with Prominent Ears
Source: Aesthetic Plast Surg. 2024 Nov 22;49(4):1054–64. doi: 10.1007/s00266-024-04478-0 (PMC11893691; doi:10.1007/s00266-024-04478-0)
Supplement: Supplementary file 1 — Supplementary file1 (DOCX 384 KB) [file 266_2024_4478_MOESM1_ESM.docx]

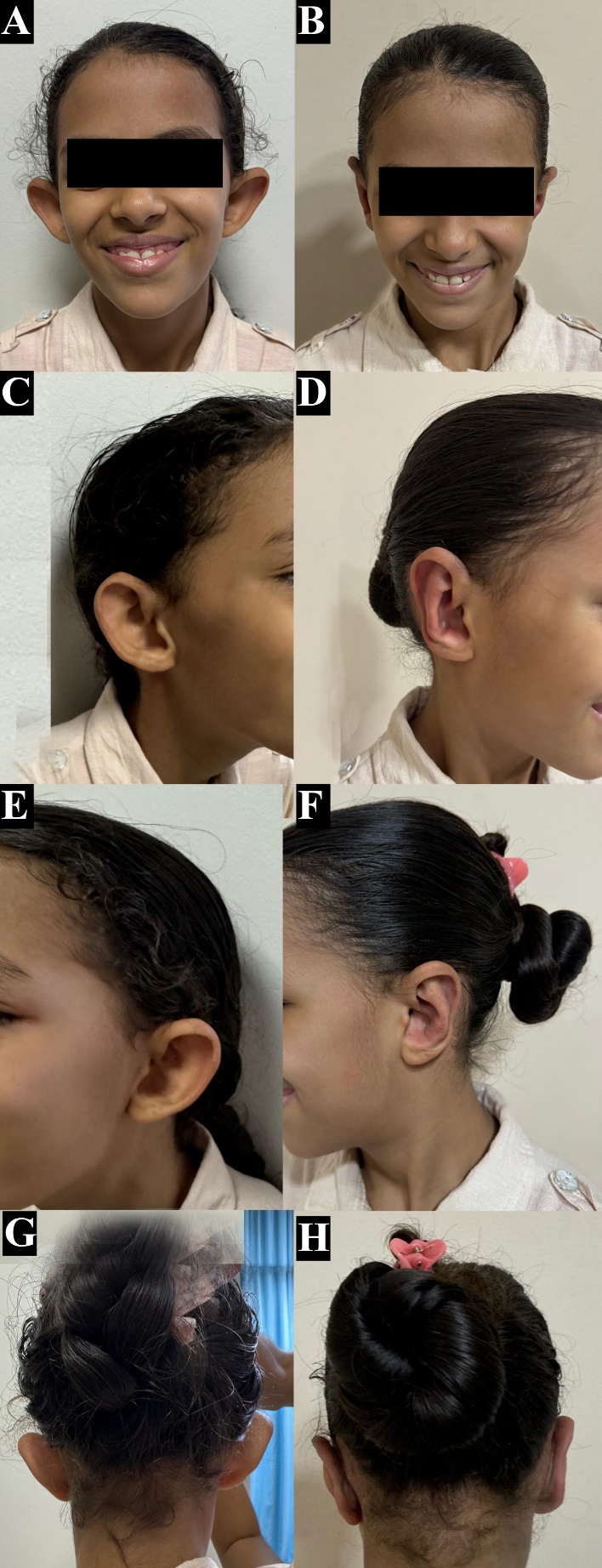


**Supplementary Fig. 1** Female patient 10 years old presented with bilateral prominent auricles. **(A) and (B)** pre- and post-operative frontal views. **(C) and (D)** pre- and post-operative oblique views of the right ear. **(E) and (F)** pre- and post-operative oblique views of the left ear. **(G) and (H)** pre- and post-operative back views.
